# Stability in ecological microcosm

**Published:** 2004-07-01

**Authors:** Yasushi Kurihara

**Affiliations:** Professor Emeritus, Tohoku University

**Keywords:** Ecological microcosm, serial transfer, parental strain, interspecific competition, directional selection, predator-mediated interaction

## Abstract

In attempt to clarify the significance of interspecific interactions in evolutionary ecology, the growth characteristics of bacterial populations sampled from ecological microcosms which act as fairly realistic models of natural ecosystems were investigated, with a particular emphasis on the ability of a system to remain reasonably stable in the genetic composition in spite of the occurrences of various mutants from native strains. Newly-emerged mutants in a community are inhibited in their multiplication, or excluded by a network of many elementary interactions between the different species of the populations, thus preserving the traits of the parental strains in a community. The interactions in the form of a network may be viewed as evidence for a maintenance of stability in a community.

## Introduction

Interactions among component species in ecological communities affect the dynamics of population densities on ecological time scales, and also influence their genetic dynamics on evolutionary time scales.1)–2) Evolutionary changes of ecological traits of species as a process of adaptive evolution involve a process in which some mutant replaces the original types within the population. This replacement can imply that some interaction(s), directly or indirectly, is actually operating between them. Out of all ecological interactions, predation and competition have received the most attention, especially for their role as a key factor determining community structure.

The term stability in ecology is frequently used to denote the ability of an ecosystem or biotic community to remain reasonably stable in form in spite of environmental changes. Because communities are subject to unpredictable variations in their physical environments and community structure, a stable community is the one that can recover its characteristic composition and relative abundance following an environmental disturbance and invasion of non-native organisms. In natural populations, however, a wide-ranging spread of genetic information may be proceeding because of their reproductive and dispersive abilities. This should lead to an increase in diversity of the strains with different genetic traits in a community, but this does not necessarily occur in stable communities. Therefore, the concept of stability should be extended to the ability to retain the genetic traits of original strains under emergence of genetic variations.

Hitherto, the overall picture of the structure and function of communities and ecosystems has come largely from the summation and integration of many separate investigations on smaller segments of these systems. The major difficulties in this approach to the understanding of communities and ecosystems are (1) the large size of such units so that only a very small portion can be studied at any one time, and (2) the almost complete absence of replicability so that few controlled experiments are possible. In this paper, a new experimental tool in ecology, the laboratory ecosystem or community, which is often called ecological microcosm or microecosystem,3)–6) has been employed in ways which have contributed significantly to the development of ecological theory. The main advantage of this systems is that the structure and activities closely parallel much larger natural systems, and that this system is replicable and controllable and thus amenable to experimentation. The objective of this study is to investigate the process of selection itself that results from predation and competition between mutants and ancestral types using microorganisms taken from the ecological microcosm, whose short generation time permit the experimental study of evolution.

## Evolutionary changes of ecological traits of bacterial populations through predator-mediated interaction

Evolutionary dynamics involving changes in both growth characteristics and population densities were studied by conducting long-term cultures of the bacterium *Escherichia coli* and the protozoan *Tetrahymena thermophila*, with a particular emphasis on “predator-mediated interaction”.7)

**Fig. 1:**
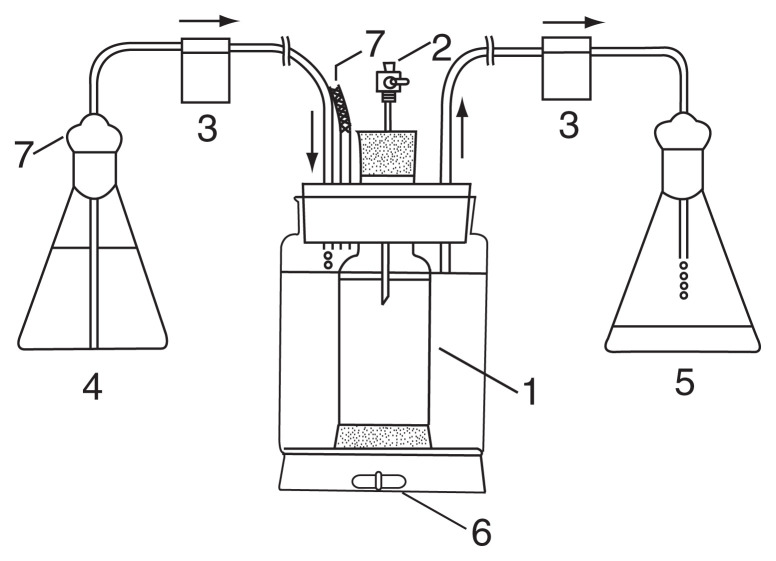
Continuous dialysis culture system. 1: dialysis chamber (cellophane tube), 2: stopcock, 3: peristaltic pump, 4: medium storage flask, 5: effluent collection flask, 6: magnetic stirrer bar, 7: cotton.7)

Continuous cultures of *E. coli*, or mixture of *E. coli* and *T. thermophila* were performed in a continuous dialysis system in which fresh medium was continuously supplied to the dialysis chamber, consisted of cellulose tubing (Visking cellulose tubing, diameter 47.6 mm) (Fig. 1). *E. coli* and *T. thermophila* coexisted over the course of the experiment. Observations of suspensions taken from the continuous mixed culture at each sampling time revealed the appearance of long-form cells of *E. coli*. Table I shows the mean cell lengths of three isolated clones with long form, compared with the original strain, at stationary phase of the cultures. The mutants with long form of *E. coli* have been reported by several workers.8)–12) The long forms have an advantage in that they are less easily eaten by feeding *T. thermophila*, namely the predation pressure of *Tetrahymena* favor long forms. One conclusion from this experiment is that predator-mediated interactions may alter the ecological trait of species.

## Ecological microcosm

Here, I would like to emphasize that *E. coli* and *T. thermophila* are those used for the purpose of experimental convenience. They are by no means biotic elements in a natural community. It is, therefore, important to examine if such phenomena mentioned above would be reproducible in microbial components in the ecological microcosm which resembles to those in nature. This is the reason why we attempted to fabricate the microcosm as an experimental model system.

Flasks containing half-strength Taub’s salt solution13) plus proteose peptone were prepared. Small amounts of an inoculum consisting of a mixture of samples taken from a pond on our university campus, a nearby oxidation pond, a paddy field and stagnant waters in the internodes of bamboo were added to a number of these flasks. The flasks were then placed in a temperature-controlled room at 24 °C under a 12:12 L/D. After two months, microscopical examination revealed that the culture media had changed into bodies of waters containing a biotic community consisting of bacteria as decomposers, *Chlorella* sp. and blue green algae as producers, and ciliate protozoa, *Cyclidium* sp., a rotifers, *Lepadella* sp. and an aquatic oligochaetes, *Aeolosoma hemprichi* as consumers. This community maintained itself for over six months.14)–15) In reference to bacterial species in this system, all bacterial colonies which appeared following plating out of high dilutions of the mature stage of microcosm were grouped into *Pseudomonas* sp., *Bacillus* sp., coryneform-type bacteria and a Gram-negative short rod, hereafter referred to as C, B, Y and E, respectively. The advantages of such microcosm are that the patterns of their metabolisms and population dynamics closely resemble much larger natural systems,4)–6),15) and that they are reproducible and controllable. Also, the systems provide a step toward clarification of the role of microbial interactions in a whole system.

## Changes of traits in a bacterial population associated with protozoal predation

In an attempt to understand the significance of predation in the changes of ecological traits of prey species, the ecological and morphological characters of bacterial species under predation by a ciliated protozoa, *Cyclidium* sp. which is a component of the microcosm were investigated. The ciliate protozoa, *Cyclidium* had been rendered bacteria-free by serial passage in the medium of penicillin, tetracycline and streptomycin. Among the 4 species of bacteria mentioned above, bacteria E was most damaged by the predation of *Cyclidium*. To see if the predator-prey relationship lead to a change in traits of bacteria E, selection was applied to batch cultures with and without *Cyclidium* by serially transferring at 7 day intervals. After 9 transfers, long cells (up to 20 μm long) appeared, together with parental short rods of 0.5–1.5 μm length, in the bacteria-protozoa population. No long forms were observed in the bacterial culture without ciliates in any of the serial transfers. This result is substantially the same as those of *E. coli*-*Tetrahymena* culture, showing that shifts in the traits are attributable to predator-prey interaction.16)

In the population subjected to strong predation, the escape abilities of the bacteria were improved by selection of elongated cells. On the other hand, evolution in the predator population of improved abilities to capture prey might not be observable after an evolutionary response in the prey that improves its ability to avoid capture. This is supported by the fact that population densities of protozoa in the culture decreased after the appearance of long type (Table II). Therefore, the coevolution ‘arms-race’, consisting of progressive adaptive improvements in the prey followed by counter-adaptations in the predator17) was not demonstrated in this prey-predator system.

## Directional selections for adhesive and dispersive traits in bacteria

The *Pseudomonas* sp. (referred to as C) possesses adhesive traits; that is, a considerable portion of biomass adheres to the bottom walls of a tube. To estimate the biomass adhering to the wall, the liquid portion of batch culture after incubation for 7 d was decanted and replaced by a salt solution. After sonicating the salt solution, the optical density was measured.

**Fig. 2:**
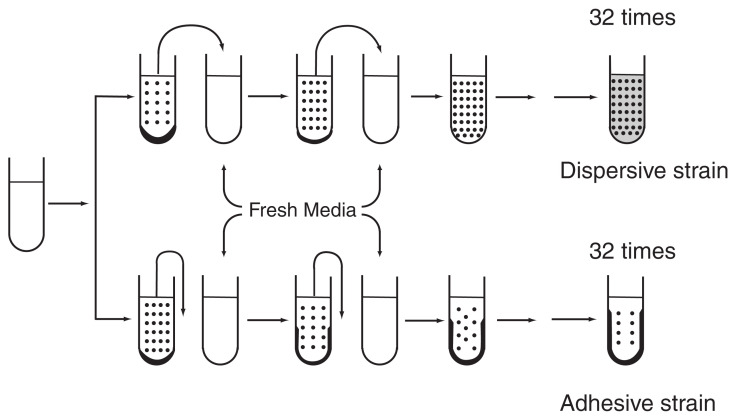
Method of directional selection.

**Fig. 3:**
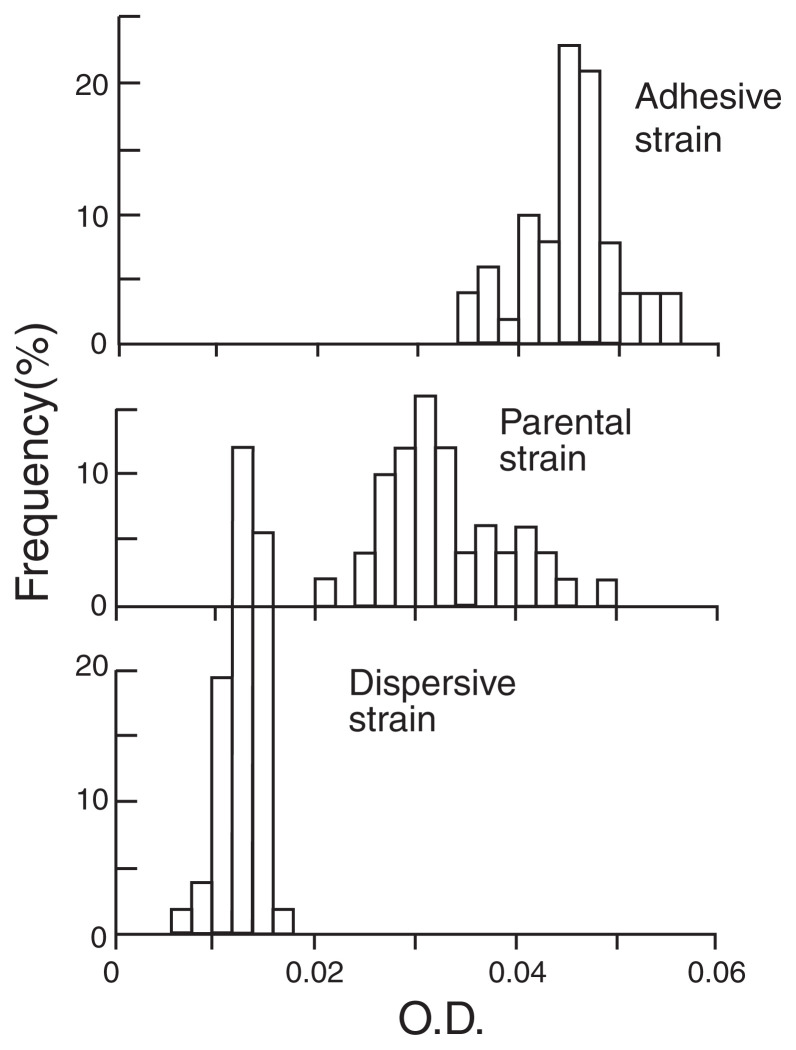
Results of directional selection for the adhesive and dispersive traits of *Pseudomonas* sp.

Two types of selections were applied to the same clone of C. In one, small amounts of the liquid portion of batch culture of C was transferred to fresh culture medium every 7 d, while in the other, the liquid portion was decanted after cultivating for 7 d and replaced by fresh culture medium. These procedures were repeated 32 times (Fig. 2). It can be seen in Fig. 3 that selection was effective in both directions and the ranges of the two selected histograms hardly overlap. The range of the histogram in the parental strain showed an intermediate position.18) This result clearly shows that strongly adhesive and dispersive strains emerged from the same clone of parental strain of C.

## Changes in specific growth rate of bacteria through intraspecies interaction

In an attempt to clarify the significance of intraspecies competition in the evolutionary changes of traits of bacteria, each of 4 kinds of bacteria from the microcosm were subjected to serial transfer at 3 d intervals when the bacterial population had attained a stationary phase. This procedure was repeated 15 times. Table III shows that selection in single-species populations under serial transfer favored an increased growth rate.19)

Repeated subculture provides an opportunity for bacteria to multiply many times. Hence mutants with various values of growth rates arise. Since mutants with higher growth rates produce more offspring, it is possible that the bacterial population could be replaced by mutants with higher growth rates in the single-species culture. These results show that intraspecies interactions alter the trait of bacteria.

## Preservation of the traits of parental strains through interaction among multispecies population

Thus, we isolated several strains which have different traits from the parental strains in the simplified subsystems which were derived from the ecological microcosm as an complex experimental system. These strains are listed as follows: long-form of E, strongly adhesive strains of C, strongly dispersive strains of C, and E, Y, B and C with higher growth rates.

Here, I would like to emphasize the fact that these strains have not yet been shown to be present in the complete microcosm composed of decomposers, primary producers and consumers, leading to the hypothesis that interactions linking multi-species populations including plants and invertebrate organisms may act to inhibit the predominance of mutants, so that parental strains can be preserved. The following experimental results support the above hypothesis.

## Preservation of parental strains, E

I had shown that appreciable numbers of long form of E occurred in the presence of ciliate protozoa, *Cyclidium*. However, we could not detect the long form in the complete microcosm containing not only *Cyclidium* but also the other components. We examined the reason why:

1. Pairwise competition experiments were performed by serially transferring the mixed culture of long form and parental strain of E at 2 d intervals in the absence of ciliate protozoa. The percentages of long form decreased in the presence of the parental strain. A selective disadvantage of the long form in competition with the parental strain may be due to a lower value for specific growth rate in the former than that in the latter strain (Table IV).16)
2. The observation of bacteria in the mixed culture with *Chlorella* showed that the normal form of E was disintegrated appreciably and the long form of E was fragmented, by the action of metabolites of *Chlorella* at the reproductive stage.
3. The metabolites of *Chlorella* also inhibit activity and number of *Cyclidium*,15),20) impeding an increase in the long form through a reduction in the number of *Cyclidium*.
4. *Aeolosoma* live in the bottom area where a large number of the long form are sedimented. This “sympatry” makes it easier for *Aeolosoma* to ingest the long form preferentially. Also, an examination of the size distribution of E in fecal pellets of *Aeolosoma* revealed that they are able to digest the long form of E. *Aeolosoma* inhibit the occurrence of the long form by their habitat and feeding habit.18)
5. *Aeolosoma* compete with *Cyclidium* for a common resources, the bacteria.15) This also acts negatively on the appearance of the long form through a reduction in the number of *Cyclidium*.
6. *Cyclidium* stimulates the bacterial floc to enlarge.21) *Cyclidium* can utilize bacterial flocs as a food source only to a small extent because the flocs are generally too large for *Cyclidium* to ingest. Therefore, *Cyclidium*, in itself, acts negatively on the appearance of the long form through a reduction in predation pressure.

## Preservation of parental strain, C

Earlier, I had shown that we could obtain strongly adhesive and dispersive strains of C by artificial selection. The values of specific growth rates of both strains in culture media are identical with each other and also with parental strain, 0.31±0.01 h^−1^ for adhesive, 0.30±0.01 h^−1^ for dispersive and 0.29±0.07 h^−1^ for parental strain. Our careful and repeated examinations revealed that dispersive and strongly adhesive strains could not be detected in the complete microcosm.

I would like to refer to the fact that there is a “partitioning of habitat” by two species of invertebrate organisms; the ciliate protozoa, *Cyclidium*, is distributed homogeneously in the liquid portion and the aquatic oligochaete, *Aeolosoma* predominates in the bottom area. Both invertebrates are bacteria feeders, though the former can swallow free-living bacterial cells through a cytostome, while the latter can lick or nibble at bacterial floc or film adhering to the bottom wall by radula-like mouth parts. Thus, there is a differentiation between the niches of the two species, allowing them to compete and yet to coexist.18)

The predation by *Cyclidium* and *Aeolosoma* will ultimately select against both types of mutant and will favor a parental strain that exhibits intermediate traits between those of both mutants (Fig. 3). Our experiment revealed that the dispersive strain in the liquid portion and the adhesive strains in the bottom area were ingested by *Cyclidium* and *Aeolosoma*, respectively, indicating that “partitioning of resources” as well as habitat by two species of invertebrates contributes to maintain the traits of parental strain in a community.

## Trade-off between interspecific competitive ability and growth rate in bacteria

I mentioned previously that selection in a single species population under serial transfer favors an increased growth rate. Now, let us examine two species populations. Serial transfers at 3 d intervals were applied to the mixed populations of E-Y, E-B, E-C, Y-B, Y-C, and B-C. Table III shows the values of specific growth rate, *r* and saturation constant, *K* of each strain sampled from single and mixed cultures that were exposed to serial transfers. It is obvious that when selection was applied to mixed populations the growth rates of both species did not change appreciably or were sometimes reduced from that of the parental strain. The reasons why bacteria under mixed-species culture conditions did not show increases in growth rate for both species were then examined by computer simulation based on the following assumptions: 1) Bacterial populations grow according to the logistic equation, and the number of bacteria in a mixed-species culture changes according to the Lotka-Volterra competition equations. 2) In two-species culture, variants of either species that have higher growth rates experience stronger competitive pressure from the second species than slower growing variants.19)

Assume that very few mutants (*N*1’) emerge from their parental strain (*N*1) which coexists with another species (*N*2). In this case, the changes in population numbers of *N*1, *N*2 and *N*1’ are calculated in accordance with formulae as follows:

$$\begin{array}{l} \frac{\text{d}N1}{\text{d}t}=r_{1}N1\frac{K1-(N1+N1^{\prime })-\alpha_{12}N2}{K1}\\ \frac{\text{d}N1^{\prime }}{\text{d}t}={r_{1}}^{\prime }N1^{\prime }\frac{K1-(N1+N1^{\prime })-\alpha_{1^{\prime }2}N2}{K1}\\ \frac{\text{d}N2}{\text{d}t}=r_{2}N2\frac{K2-(N2)-\alpha_{21}(N1+N1^{\prime })}{K2} \end{array}$$

*r*_i_ : the specific growth rate of i strain
*K*_i_ : the saturation density of i strain
*α*_ij_ : the competition coefficient for the effect of j strain on i strain

which are modified from the Lotka-Volterra competition equations.22) Rarefactions are made on *N*1, *N*2 and *N*1’ every 3 d of cultivation. These procedures follow the experimental protocol.19)

**Fig. 4:**
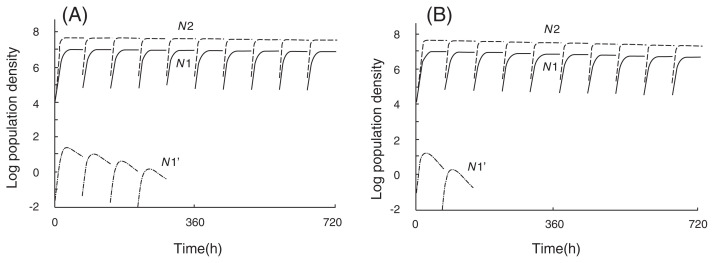
Computer simulations of *N*1, *N*2, and *N*1’ in a mixed culture of *N*1 and *N*2, where the mutant (*N*1’) with a higher growth rate emerges from *N*1. The values of growth rates and the saturation densities of *N*1 and *N*2 used for the calculation are the same as those of the parental strains of species Y and E in Table III; *r*_1_ = 0.366, *r*_2_ = 0.549, *K*_1_ = 2.03 × 10^7^ and *K*_2_ = 4.95 × 10^7^. The competition coefficients *α*_12_ and *α*_21_ are 0.2 and 0.01, which were estimated from the population changes in mixed cultures of two bacteria using the competition equation. (A) If it is assumed that the growth rate of *N*1’ is 0.448 and *α*_1’2_ is 0.23, *N*1’ is extinct at 288 h. (B) If it is assumed that *α*_1’2_ is 0.25, *N*1’ is extinct at 144 h.19)

**Fig. 5:**
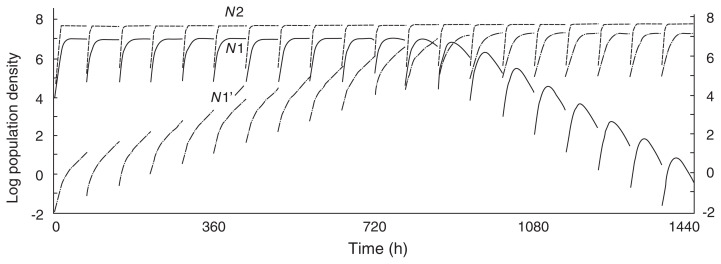
Computer simulations of *N*1, *N*2, and *N*1’ in a mixed culture of *N*1 and *N*2 where the mutant (*N*1’) with a lower growth rate emerges from *N*1. The parameters for growth rates, saturation constants, and competition coefficients of *N*1 and *N*2 are the same as those in Fig. 4. If it is assumed that the growth rate of *N*1’ is 0.217 and the competition coefficient *α*_1’2_ is 0.1, the mutant increases its cell density with time after transfer, and eventually excludes its parental strain (*N*1).19)

Figure 4 shows the simulated changes in numbers of *N*1, *N*2 and *N*1’ which is the mutant of *N*1 and which has a higher growth rate than *N*1. During the process, *N*1’ is excluded due to rarefaction and to the competitive pressure from *N*2 because the competition between *N*1’ and *N*2 should be severe as described in the assumption mentioned above. This may be an explanation for the experimental results (Table III) showing that the growth rates of E (coexisting with B or C), Y (coexisting with B or C), B (coexisting with Y), and C (coexisting with E, Y, or B) are nearly the same as those of the respective parental strains. If the growth rate of mutant (*N*1’) is lower than that of the parental strain (*N*1) and the competition between *N*1’ and *N*2 is not severe, *N*1’ increase in density until it eventually excludes the parental strain (Fig. 5). This may be an explanation for the experimental results (Table III) showing that the growth rates of E (coexisting with Y), Y (coexisting with E) and B (coexisting with E) are all lower than those of the respective parental strains.

The result that a mixed population exposed to serial transfer did not lead to increased growth rates of both species supports the contention that adaptations to interspecies competition are superior to intraspecies competition in the mixed culture. Accordingly, interspecies competition would act to inhibit the proliferation of mutants with higher growth rate, thus preserving the traits of the parental strains.

Our results may be viewed as evidence for a tradeoff between interspecific competitive ability and growth rate, that is, adaptation to a higher growth rate reduces interspecific competitive ability. One explanation as to why fast-growing mutants may be competitively inferior in mixed culture (Fig. 4) may be that growth and reproduction of such mutants are particularly prone to interference from other species, including the negative action of metabolites produced by other species. This interpretation is consistent with Luckinbill’s findings, obtained with six ciliate species, that the relative competitive abilities of ciliate species were negatively correlated with specific growth rate, *r* and saturation constant, *K*.23)

It is unclear why and how mutants having lower growth rates could exclude parental strains in mixed culture (Fig. 5). One possibility is that mutants having lower growth rates may be competitively superior in mixed culture. Also it is possible that a resource substance which may be used by the mutant with lower *r* is produced as a by-product of other species’ cell metabolism.24)

## Conclusion and perspective

Ecosystems or biotic communities show stability in the sense that they tend to shift back toward their original states in biotic elements and relative abundances, once the external disturbance, such as changes in abiotic environments and invasions by non-native organisms, has ceased. However, it should not be forgotten that a community undergoes internal shifts by the emergence of various mutants. I have seen that a variety of interactions can influence community stability. Yet, it has to be admitted that precise statements as to their relative potencies must await further advances in our knowledge and understanding. Nevertheless, certain tentative conclusions and future perspective can be drawn.

Some ecologists have presented evidence to show that stability is greater in more diverse systems.25),26) Outbreaks of mutants are common in simple systems. It is known that mono-culture systems of bacterial species are sometimes replaced by newly-emerged mutants. On the other hand, such outbreaks seem very uncommon in the complex systems. One basis for the greater stability in more diverse system would be that the network of interactions inhibits outbreaks of mutants, thus preserving the traits of parental species.

A connection between diversity and stability is attractive, for it provides a practical argument against the extreme simplification of ecosystems favored by recent technology. In connection with this, I would like to point out that for the introduction of R-DNA-engineered microorganisms into the simple mono-culture systems favored by modern agriculture, it cannot be said that all ecological communities are stable and resilient to perturbation.

To our knowledge, it has been considered that major shifts in microbial communities mainly reflect alterations in environmental factors, that is, population shifts are generally attributable to selection by environmental factors. However, our results show that major shifts may reflect the interaction in a community. The evaluation of the risk associated with a particular introduction should be based on the properties of the engineered organism and the interactions in microbial communities in its target environment.

In addition to the study of the basic principles of ecology,27) the ecological microcosm may also become a very valuable diagnostic tool.4),28) For example, new R-DNA-engineered organisms developed in the advanced countries and many new organic compounds are released to the environment before we understand all of the possible effects of them. The usual procedure, if any testing is done at all, is to do toxicity tests on one or more vertebrate species. Unfortunately, such tests are relatively meaningless since new creatures and pollutants are applied, in practice, to the community and ecosystem levels of organization and not to these organism levels. Laboratory ecosystems will make excellent devices for the examination of the short and long-range effects of potential pollutants on the actual level of biological organization to which they are applied.29)–32) Such studies can be carried out before the potential pollutants are released.

## Figures and Tables

**Table I:** Cell lengths (μm) of the original strain and long-form clones isolated from the mixed culture of *E. coli* and *T. thermophila*. The number of individuals counted (n) are shown at right.

Strain	Mean±S. D.	(n)
Original	1.43±0.23	(60)
Long-form clone 1	6.13±7.40	(45)
2	4.14±2.62	(47)
3	6.67±5.12	(43)

**Table II:** The mean density of *Cyclidium* in the 7 day culture from the first through the seventh transfer (before the appearance of long type) and that in the 7 day culture from the eighth through the twenty-eighth transfer (after the appearance of long type)

Number of transfers	*Cyclidium* (no./ml)		

	Mean	SE	n
1–7	27,000	1,740	7
8–28	20,700	811	21

Significant differences (*P* < 0.01) between two means.

**Table III:** Specific growth rates and saturation constants of strains under serial transfer at 3 d intervals and those of parental strains (mean±SE, *n* = 5)

Bacterium	Strain	Specific growth rate, *r* (h^−1^)	Saturation constant, *K* (10^7^ cells/mL)
E	Parental	.549±.010	4.95±.32
	Single	.614±.023*	3.75±.10*
	Mixed with Y	.498±.019*	4.40±.13
	B	.573±.012	3.92±.09*
	C	.576±.023	4.24±.28
Y	Parental	.366±.009	2.03±.11
	Single	.448±.026*	2.35±.11
	Mixed with E	.217±.023*	1.94±.05
	B	.311±.026	3.82±.31*
	C	.381±.009	2.49±.20
B	Parental	.654±.024	.248±.013
	Single	.704±.011	.215±.006
	Mixed with E	.533±.037*	.250±.015
	Y	.702±.046	.223±.008
	C	.730±.013*	.119±.016*
C	Parental	.253±.017	6.77±.62
	Single	.288±.006	6.55±.37
	Mixed with E	.247±.004	7.39±.42
	Y	.253±.005	7.52±.57
	B	.249±.009	7.75±.40

* Significant differences (*P* < .05) between parental and selected strains.

**Table IV:** Specific growth rates and saturation constants of selected and parental strains (mean±SE)

Strain	Specific growth rate (hours^−1^)	Saturation constant (10^7^ cells/ml)
Parental	0.427±0.009	3.50±0.16
Long form (selected)	0.282±0.013*	0.78±0.07*

* Significant differences (*P* < 0.01) between parental and selected strains.

## References

[1] Pimentel D. (1961) On a genetic feed-back mechanism regulating populations herbivores, parasite and predators. Am. Nat. 95, 65–79.

[2] Pimentel D. Stone F. A. (1968) Evolution and population ecology of parasite-host systems. Can. Entomol. 100, 655–662.

[3] Kurihara Y. (1960) Biological analysis of the structure of microcosm with special reference to the relations among biotic and abiotic factors. Sci. Rep. Tôhoku Univ. Ser. IV (Biol.) 26, 269–296.

[4] Cooke G. D. (1971) Aquatic laboratory microsystems and communities. In The Structure and Function of Freshwater Microbial Communities (ed. Cairns J. Jr.). University Press of Virginia, Charlottesville, Virginia, pp. 47–85.

[5] Odum E. P. (1971) Fundamentals of Ecology. Saunders, Philadelphia.

[6] Beyers R. J. Odum H. T. (1993) Ecological Microcosms. Springer-Verlag, New York.

[7] Nakajima T. Kurihara Y. (1994) Evolutionary changes of ecological traits of bacterial populations through predator-mediated competition 1. Experimental analysis. Oikos 71, 24–34.

[8] Normark S. Boman H. Matsson E. (1969) Mutant of *Escherichia coli* with anomalous cell division and ability to decrease episomally and chromosomally mediated resistance to ampicillin and several other antibiotics. J. Bacteriol. 97, 1334–1342. 4887513 10.1128/jb.97.3.1334-1342.1969 PMC249852

[9] Normark S. (1970) Genetics of a chain-forming mutant of *Escherichia coli*. Transduction and dominance of the envA gene mediating increased penetration to some antibacterial agents. Genet. Res. 16, 63–78. 4922970 10.1017/s0016672300002287

[10] Bachmann B. J. (1987) Linkage map of *Escherichia coli* K-12, ed. 7. In *Escherichia coli* and *Salmonella typhimurium*—Cellular and Molecular Biology. Vol. II (eds. Neidhardt F. C. Ingraham J. L. Low K. B. Magasanik B. Schaechter M. Umbarger H. D.). American Society for Microbiology, Washington, D. C., pp. 807–876.

[11] Adler H. I. Hadigree A. A. (1964) Analysis of a gene controlling cell division and sensitivity to radiation in *Escherichia coli*. J. Bacteriol. 87, 720–726. 14127589 10.1128/jb.87.3.720-726.1964 PMC277076

[12] Talor A. L. Trotter C. D. (1972) Linkage map of *Escherichia coli* strain K-12. Bacteriol. Rev. 36, 504–524. 4568762 10.1128/br.36.4.504-524.1972 PMC408330

[13] Taub F. B. Dollar A. M. (1964) A *Chlorella-Daphnia* food chain study; the design of a compatible chemically defined culture medium. Limnol. Oceanogr. 9, 61–74.

[14] Kurihara Y. (1978) Studies of “succession” in a microcosm. Sci. Rep. Tôhoku Univ. Ser. IV (Biol.) 37, 151–160.

[15] Kurihara Y. (1978) Studies of the interaction in a microcosm. Sci. Rep. Tôhoku Univ. Ser. IV (Biol.) 37, 161–177.

[16] Shikano S. Luckinbill L. S. Kurihara Y. (1990) Changes of traits in a bacterial population associated with protozoal predation. Microb. Ecol. 20, 75–84. 24193965 10.1007/BF02543868

[17] Abrams P. A. (1986) Is predator-prey coevolution an arms race? Tree 1, 108–110. 21227793 10.1016/0169-5347(86)90037-6

[18] Kurihara Y. (1989) Interaction and stability of microbial communities in experimental model systems. In Recent Advances in Microbial Ecology (eds. Hattori T. Ishida Y. Maruyama Y. Morita R. Y. Uchida A.). Japan Scientific Societies Press, Tokyo, pp. 11–20.

[19] Kurihara Y. Shikano S. Toda M. (1990) Trade-off between interspecific competitive ability and growth rate in bacteria. Ecology 71(2), 645–650.

[20] Kawabata Z. Kurihara Y. (1978) Computer simulation study on the relationships between the total system and subsystems in the early stages of succession of the aquatic microcosm. Sci. Rep. Tôhoku Univ. Ser. IV (Biol.) 37, 179–204.

[21] Suzuki T. Kurihara Y. (1981) The role of bacterial flocs in the population dynamics of bacteria and protozoa in continuous cultures. Jpn. J. Ecol. 31, 23–29.

[22] Gause G. F. (1934) The Struggle for Existence. Williams & Wilkins, Baltimore, Maryland.

[23] Luckinbill L. S. (1979) Selection and the r/K continuum in experimental populations of protozoa. Am. Nat. 113, 427–437.

[24] Levin B. R. (1972) Coexistence of two asexual strains on a single resource. Science 175, 1272–1274. 4551427 10.1126/science.175.4027.1272

[25] Elton C. (1958) The Ecology of Invasions by Animals and Plants. Methuen, London.

[26] MacArthur R. H. (1972) Geographical Ecology. Harper and Row, New York.

[27] Sugiura K. Kawasaki Y. Kinoshita M. Murakami A. Yoshida H. Ishikawa Y. (2003) A mathematical model for microcosms: formation of the colonies and coupled oscillation in population densities of bacteria. Ecological Modelling 168, 173–201.

[28] Sugiura K. (1998) A materially-closed aquatic-ecosystem: A useful tool for determining changes of ecological processes in space. Biol. Sci. Space 12(2), 115–118. 11541877 10.2187/bss.12.115

[29] Sugiura K. (2001) Effects of Al^3+^ ions and Cu^2+^ ions on microcosms with three different biological complexities. Aquatic Toxicology 51, 405–417. 11090899 10.1016/s0166-445x(00)00126-0

[30] Sugiura K. (1996) The use of an aquatic microcosm for pollution effects assessment. Wat. Res. 30(8), 1801–1812.

[31] Inamori Y. Murakami K Sudo R. Kurihara Y. Tanaka N. (1992) Environmental assessment method for field release of genetically engineered microorganisms using microcosm systems. Water Sci. Tech. 26(9–11), 2161–2164.

[32] Murakami K. Inamori Y. Sudo R. Kurihara Y. (1992) Effect of temperature on prosperity and decay of genetically engineered microorganisms in a microcosm system. Water Sci. Tech. 26(9–11), 2165–2168.

